# Unilateral sectioning of the superior ovarian nerve of rats with polycystic ovarian syndrome restores ovulation in the innervated ovary

**DOI:** 10.1186/1477-7827-8-99

**Published:** 2010-08-19

**Authors:** Leticia Morales-Ledesma, Rosa Linares, Gabriela Rosas, Carolina Morán, Roberto Chavira, Mario Cárdenas, Roberto Domínguez

**Affiliations:** 1 Biology of Reproduction Research Unit, Physiology of Reproduction Laboratory FES Zaragoza, UNAM, AP 9-020, CP 15000, México, DF, México; 2 Departament of Biology and Toxicology of Reproduction, Science Institute BUAP, México; 3 Instituto Nacional de Ciencias Médicas y de la Nutrición "Salvador Zubirán", México

## Abstract

The present study tested the hypothesis that if polycystic ovary syndrome (PCOS) results from activating the noradrenergic outflow to the ovary, unilaterally sectioning the superior ovarian nerve (SON) will result in ovulation by the denervated ovary, and the restoration of progesterone (P4), testosterone (T) and estradiol (E2) normal serum level. A single 2 mg dose of estradiol valerate (EV) to adult rats results in the development of a syndrome similar to the human PCOS. Ten-day old rats were injected with EV or vehicle solution (Vh) and were submitted to sham surgery, unilateral or bilateral sectioning of the SON at 24-days of age. The animals were sacrificed at 90 to 92 days of age, when they presented vaginal estrus preceded by a pro-estrus smear. In EV-treated animals, unilateral sectioning of the SON restored ovulation by the innervated ovary and unilateral or bilateral sectioning of the SON normalized testosterone and estradiol levels. These results suggest that aside from an increase in ovarian noradrenergic tone in the ovaries, in the pathogenesis of the PCOS participate other neural influences arriving to the ovaries via the SON, regulating spontaneous ovulation. Changes in P4, T and E2 serum levels induced by EV treatment seem to be controlled by neural signals arising from the abdominal wall and other signals arriving to the ovaries through the SON, and presents asymmetry.

## Background

The polycystic ovarian syndrome (PCOS) affects approximately 3% to 5% of the female population [1] and is the most common cause of infertility in women of reproductive age [2-4]. PCOS is a complex disease characterized by ovulatory failure, the presence of ovarian cysts, menstrual irregularities, amenorrhea, hyperandrogenism and variable levels of circulating gonadotropins [3,5]. Commonly, PCOS is accompanied by obesity, hirsutism, and, in the vast majority of cases, infertility [4]. PCOS may lead to complications involving glucose metabolism, dyslipidemias, cardiovascular disease and cancer [1,2]. In women with PCOS, lowering estradiol levels treatments with clomiphen citrate [4,6], bilateral extirpation of a piece of the ovaries [5,7], or by electro acupuncture treatment [8], restores ovulation and menstrual cycles.

Treating new-born rats with testosterone propionate (TP) or progesterone (P4) resulted in endocrine and histological disorders, similar to those found in women with PCOS syndrome [9]. However, data from TP-androgenized rats showed that serum luteinizing hormone (LH), follicle stimulating hormone (FSH), testosterone (T) and estradiol (E2) concentrations were similar to those in the control group [10]. Previously, we showed that anovulatory syndrome induced by TP does not occur if the animals had peripheral noradrenergic denervation induced by guanethidine treatment [11].

Estradiol valerate (EV) is a long-acting estrogen, and a single 2 mg dose to adult rats results in the development of a syndrome characterized by loss of estrous cyclicity, persistent vaginal cornification, anovulation, formation of follicular cysts, alterations in basal and pulsatile LH and FSH concentrations, and high concentrations of E2. These symptoms are considered similar to human PCOS [12-16]. EV treatment to pre-pubertal female rats results in higher E2 levels in serum, lower gonadotropins and androstenedione concentrations in serum, and an alteration in the follicular growth dynamic that favors the formation of follicular cysts [17]. At the ovarian level, EV treatment results in a higher capacity of the ovarian nerve terminals to incorporate and release norepinephrine (NE), increases in ovarian NE content and lower ovarian β-adrenergic receptor number in the ovarian compartments [12,13,16,17].

The bilateral sectioning of the superior ovarian nerve (SON) to pre-pubertal and adult EV-treated rats restores the estrous cycle and ovulation [13,17]. Based on these and other results it has been proposed that a derangement in neurogenic inputs may contribute to the development of ovarian pathologies like the PCOS [13,16,17].

Ovulation and hormone secretion by the ovaries are regulated by hormonal signals secreted by the hypothalamic-pituitary-ovarian axis, and neural influences modulating the ovarian response to such hormonal signals [16,18,19].

The SON and the ovarian plexus nerve provide noradrenergic ovarian innervations [20,21]. The bilateral sectioning of the SON did not modify ovulation nor gonadotropins levels; but compared to control groups lowered E2 and P4 serum levels [20,22-24]. On the hand, unilateral sectioning of the SON results in a significantly lower ovulation capacity by the denervated ovary [23,24], which is not restored by gonadotropins treatment [25].

In the present study we tested the hypothesis that if PCOS results from the activation of noradrenergic outflow to the ovary, unilateral sectioning the SON will restore ovulation by the denervated ovary, without modifying the lack of ovulation by de innervated one. In addition, alterations to the estrous cycle, as well as P4, T and E2 serum concentrations, will be normalized by unilateral ovarian denervation.

## Methods

All experiments were carried out in strict accordance with the Mexican Law of Animal Treatment and Protection Guidelines. The Committee of the Facultad de Estudios Superiores Zaragoza approved the experimental protocols.

The study was performed using pre-pubertal female rats of the CIIZ-V strain from our own breeding stock. Animals were maintained under controlled lighting conditions (lights on from 05:00 to 19:00 h); with free access to rat chow and tap water. The number of animals in each group is indicated in table 1.

### Animal treatment

Ten days old rats were injected a single dose a 0.1 ml of corn oil (vehicle Vh) or with 2 mg of EV (Sigma Chem. Co., St. Luis, Mo. USA) dissolved in 0.1 ml of corn oil. When the animals reached 24 days of age, they were allotted to one of the following groups:

Rats injected with Vh: a) with unilateral section of the SON; b) with bilateral section of the SON; c) left untouched until sacrifice.

Rats injected with EV: a) with unilateral section of the SON; b) with bilateral section of the SON; c) left untouched until sacrifice.

**Figure 1:**
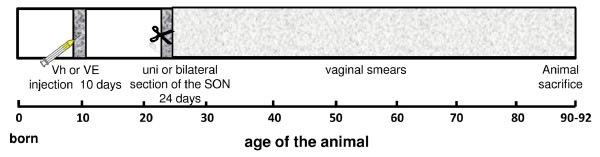
Experimental design.

Sectioning of the SON and sham surgery procedures were performed between 10:00 and 12:00 h, following previously described methodology [17,23-25]. In brief, animals were anesthetized with ether, a unilateral or bilateral dorso-lateral incision, including skin and muscle, was performed and the wound was subsequently sealed. For sectioning the SON, the animals were anesthetized with ether, a unilateral or bilateral dorso-lateral incision, including skin and muscle, was performed, and one or both ovaries were exposed. With the aid of fine forceps, the ovarian ligament was sectioned at approximately 1 cm from the ovary. The ovary was subsequently returned to the abdominal cavity and the wound sealed. The age of first vaginal estrus (puberty), was recorded and daily vaginal smears were taken thereafter. All the animals were sacrificed at 90-92 days of age, presenting vaginal estrus preceded by a pro-estrus smear (Figure 1).

### Autopsy procedures

Animals were sacrificed by decapitation between 10.00 AM and noon. The blood from the trunk was collected, allowed to clot, and centrifuged during 15 min at 3,000 RPM. The serum was stored at -20°C, until P4, T, E2, FSH and LH levels were measured. At autopsy, the oviducts were dissected and the number of ova counted with the aid of a dissecting microscope, as previously [24-26]. The free movement of the ovary in the abdominal cavity was used to verify the complete sectioning of the suspensor ligament. The ovaries were excised and processed for NE measurement as it was previously described [27]. In brief, each ovary was homogenized in 300 μl of 0.1 M perchloric acid containing dihydroxy-benzylamine as internal standard. NE was extracted from homogenates by absorption in acid-washed alumina, and measured using HPLC [27]. The samples were assayed in 50 μl duplicated aliquots. The sensitivity of the method was 0.1 ng.

### Hormone measurement

Serum concentration of E2 (pg/ml), T (ng/ml) and P4 (ng/ml) were measured using radioimmunoassay, with kits purchased from Diagnostic Products (Los Angeles, CA, USA). The intra- and interassay coefficients of variation were 8.35% and 9.45% for P4, 8.12% and 9.28% for E2, and 9.65% and 10.2% for T respectively. FSH and LH (ng/ml) levels in serum were measured by the double antibody RIA technique, employing reagents and protocols kindly supplied by the NIADDK National Pituitary Program (Bethesda, MD, USA). Intra- and inter-assay variations were in the order of 5.1% and 6.5% for LH, and 4% and 7.9% for FSH. The results are expressed in terms of NIADDK standards RP-2.FSH and LH.

### Statistical analysis

Data on NE, P4, T, E2, FSH and LH concentrations, were analyzed by multivariate analysis of variance (MANOVA), followed by Turkey's test. The age of first vaginal oestrous and the number of ova shed by ovulating animals was analyzed using Kruskal-Wallis test, followed by Mann-Whitney U-test. Mann-Whitney U-test or Student's t test were used for comparing the results of two groups. The ovulation rate (number of ovulating animals/number of treated one) was analyzed using Fisher"s exact probability test or the Chi square test. A p-value of less than 0.05 was considered significant.

## Results

Compared to Vh-injected animals, EV treatment advanced the age of first vaginal estrus (FVO) (Table 1). Most rats injected with EV were in constant estrus.

FVO was not modified by unilateral or bilateral sham surgery or sectioning of the SON in Vh-injected animals. Right or bilateral sham surgery to EV-treated rats and left, right or bilateral sectioning of the SON delayed FVO in comparison with untouched EV-treated ones (Table 1).

**Table 1:** Mean ± SEM puberty in rats injected with vehicle (Vh) or estradiol valerate on day 10 of age

Group	n	FVO	Group	n	FVO
**Vh**	9	37.8 ± 0.9	**EV**	11	27.0 ± 0.5
**Vh+left-sham**	8	38.1 ± 0.8	**EV-LS**	8	28.3 ± 0.4
**Vh+LSON**	10	37.8 ± 0.4	**EV-LSON**	9	36.1 ± 0.8*
**Vh+right sham**	9	36.9 ± 0.5	**EV-RS**	7	33.4 ± 0.9*
**Vh+RSON**	11	37.5 ± 0.5	**EV-RSON**	10	33.0 ± 1.4*
**Vh+bilateral sham**	9	38.1 ± 0.4	**EV-BS**	7	32.0 ± 0.3*
**Vh+BSON**	8	37.2 ± 0.7	**EV-BSON**	9	35.8 ± 1.5*

*p < 0.05 *vs. *EV group (Kruskal-Wallis, followed by Mann-Whitney U-test)

FVO (age of first vaginal oestrus; LSON (section of the left superior ovarian nerve); RSON (section of the right); BSON (bilateral section)

EV-treated rats presented regular estrous cycles after unilateral or bilateral sectioning of the SON. The Vh-injected rats showed normal cycles.

None of the EV treated rats ovulated (0/11), while 9/9 of Vh-injected ones did (p < 0.01 Fisher's exact probability test). Unilateral or bilateral sham operation to Vh or EV treated animals did not modify spontaneous ovulation (for Vh-injected 24/26 vs. 9/9, non significant (n.s.); for EV injected 5/22 vs. 0/11 ovulated, n.s.; chi square test).

Bilateral sectioning of the SON to Vh-injected animals did not modify the spontaneous ovulation rate nor the number of ova shed (8/8 vs. 9/9 n.s. Fisher's exact probability test; 10.3 ± 1.3 vs. 7.0 ± 1.3, n.s. Mann-Whitney U-test). Bilateral sectioning of the SON to EV-treated animals restored ovulation in a similar way that observed in bilaterally denervated rats injected with Vh (ovulation rate EV+BSON 5/9 vs. Vh+BSON 8/8; n.s. Fisher's exact probability test; number of ova shed 6.6 ± 1.6 vs. 10.3 ± 1.3, n.s. Mann-Whitney U-test).

Taken together, the unilateral sectioning of the SON (right or left section) to EV-treated rats resulted in higher spontaneous ovulation by the innervated ovary than in rats injected with EV [16/19 vs. 0/11, p < 0.01 (chi square test)].

**Figure 2:**
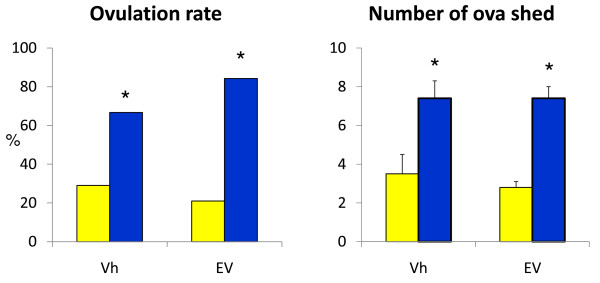
**Ovulation rate in rats with unilateral section of the SON**. Percent of ovulating rats and mean ± SEM number of ova shed in rats injected with vehicle (Vh) or estradiol valerate (EV) at 10 days of life, submitted to unilateral section of the superior ovarian nerve, sacrificed at day 90-92 of life. Ovulation rate * p < 0.05 vs. denervated (Fisher's test) Number ova shed * p < 0.05 vs. denervated (kruskal-Wallis test)

Rats treated with Vh or EV submitted to unilateral sectioning of the SON, showed higher ovulation rates and number of ova shed by the innervated ovary than by the denervated ovary (Figure 2).

Table 2 presents the hormone concentrations results of Vh or EV injected animals with bilateral sectioning of the SON. In Vh-injected animals, bilateral sham surgery did not modify P4, T and E2, serum levels, while the bilateral sectioning of the SON resulted in lower hormone levels than in animals with sham surgery.

**Table 2:** Mean ± SEM of hormone levels in vehicle (Vh) or estradiol valerate (EV) injected on day 10 of age

Group	Progesterone (ng/ml)	Testosterone (ng/ml)	Estradiol (pg/ml)
**Vh**	11.4 ± 1.4	25.0 ± 6.1	21.2 ± 1.3
**Vh+Sham bilateral**	13.9 ± 1.3	16.1 ± 0.5	22.1 ± 1.4
**Vh+BSON**	9.0 ± 1.4†	ND*†	15.0 ± 1.9†
**EV**	13.9 ± 2.4	54.5 ± 5.7	36.7 ± 5.5
**EV+Sham bilateral**	10.9 ± 0.9	32.1 ± 6.2§	18.7 ± 2.0§
**EV+BSON**	6.6 ± 0.9#§	27.8 ± 4.6§	18.7 ± 1.6§

† p < 0.05 vs. Vh+sham bilateral operated group (ANOVA followed by Tukey Test)

§ p < 0.05 vs. EV group (ANOVA followed by Tukey Test)

* p < 0.05 vs. Vh group (ANOVA followed by Tukey Test)

# p < 0.05 vs. their EV+sham bilateral operated group (Student's "t" test)

Compared with untouched EV-treated animals bilateral-sham surgery or sectioning of the SON resulted in lower T and E2 serum levels. P4 levels were lower in EV-treated rats with bilateral section of the SON than in EV-treated animals with bilateral sham operation.

Compared with untouched Vh-treated, animals EV treatment resulted in higher T and E2 serum concentration. P4 concentration differences were not observed between these two groups (Table 3).

**Table 3:** Mean ± SEM of hormone levels in animals injected with vehicle (Vh) or estradiol valerate (EV) and unilateral sectioning of the SON

Group	Progesterone (ng/ml)	Testosterone (ng/ml)	Estradiol (pg/ml)
**Vh**	11.4 ± 1.4	25.0 ± 6.1	21.2 ± 1.3
**EV**	13.9 ± 2.4	54.5 ± 5.7*	36.7 ± 5.5*
**Vh+ left-sham**	15.9 ± 1.8	37.5 ± 3.4	22.8 ± 2.62
**Vh+LSON**	8.1 ± 1.7†	16.0 ± 1.8†	23.1 ± 2.9
**EV+ left-sham**	9.9 ± 0.9	29.1 ± 0.3§	16.3 ± 1. 6§
**EV+LSON**	16.6 ± 1.9#	17.8 ± 2.3#	15.9 ± 1.2§
**Vh+ right-sham**	11.5 ± 1.8	36.04 ± 7.2	21.6 ± 1.7
**Vh+RSON**	13.8 ± 1.5	40.9 ± 9.9	14.1 ± 1.8†
**EV+ right-sham**	12.3 ± 1.8	35.5 ± 12.8	20.7 ± 3.2§
**EV+RSON**	5.2 ± 0.6#	20.8 ± 4.4	18.1 ± 1.9§

* p < 0.05 vs. Vh group (Student's "t" test)

† p < 0.05 vs. their Vh+sham operated group (Student's "t" test)

# p < 0.05 vs. their EV+sham operated group (Student's "t" test)

§ p < 0.05 vs. EV group (MANOVA followed by Tukey's Test)

Compared with untouched Vh-treated animal left or right sham surgery to Vh-injected animals did not modify P4 levels. In comparison with Vh-injected sham-operated rats, sectioning the left SON resulted in lower P4 levels.

Sham operation to EV-treated animals did not modify P4 levels in comparison with EV-treated rats. Sectioning the left SON resulted in higher P4 levels than in EV-sham operated animals, while in the right SON-sectioned animals an inverse response occurred.

Sham operation to Vh-injected animals did not modify T levels, while sectioning the left SON resulted in lower hormone levels than in sham-operated rats.

In EV-treated animals, left sham-surgery resulted in significantly lower T levels when compared to with EV-treated ones left untouched, while the section of the left SON resulted in lower T levels than in EV-sham operated ones.

In Vh-injected animals, unilateral sham surgery did not modify E2 serum levels, while sectioning the right SON resulted in lower hormone levels than in animals with sham-surgery.

In EV-treated animals, unilateral sham surgery and unilateral section of the SON resulted in lower E2 levels.

### FSH and LH levels

No significant differences in LH levels were observed. In rats injected with EV, sectioning the L-SON resulted in lower FSH levels than in Vh-treated rats, while bilateral sectioning of the SON resulted in higher levels of the FSH than in EV-injected rats (Table 4).

**Table 4:** Mean ± SEM of FSH and LH (ng/ml) levels in animals injected with vehicle (Vh) or estradiol valerate (EV) and unilateral or bilateral sectioning of the SON

Group	FSH	LH
**Vh**	6.1 ± 0.7	0.56 ± 0.11
**EV**	4.0 ± 0.7	0.38 ± 0.07
**Vh+left sham**	8.0 ± 0.8	0.44 ± 0.05
**Vh+LSON**	7.4 ± 0.5	0.40 ± 0.06
**EV+left sham**	4.0 ± 0.7	0.38 ± 0.07
**EV+LSON**	4.4 ± 0.2	0.56 ± 0.12
**Vh+right sham**	8.3 ± 1.3	0.43 ± 0.08
**Vh+RSON**	6.7 ± 1.6	0.47 ± 0.06
**EV+right sham**	3.4 ± 0.4	0.43 ± 0.02
**EV+RSON**	4.4 ± 0.5	0.45 ± 0.07
**Vh+bilateral sham**	5.9 ± 0.5	0.6 ± 0.06
**Vh+BSON**	5.3 ± 1.8	0.59 ± 0.08
**EV+bilateral sham**	4.2 ± 0.5	0.40 ± 0.05
**EV+BSON**	8.2 ± 1.5#¥	0.39 ± 0.05

# p < 0.05 vs. EV group

¥p < 0.05 vs. EV+bilateral sham (Student's t test)

### NE ovarian levels

NE levels were higher in both ovaries of EV-treated animals than in Vh-treated rats. Sectioning the left SON resulted in lower NE levels in the left ovary (denervated). Sectioning the right ovary resulted in lower NE levels in both ovaries. The bilateral sectioning of the SON resulted in lower NE levels in both ovaries (Table 5).

**Table 5:** Mean ± SEM of norepinephrine concentration (pg/mg of the ovary) in animals injected with estradiol valerate (EV) and unilateral or bilateral sectioning of the SON

Group	Left Ovary	Right Ovary
**Control**	405.5 ± 74	457.7 ± 76
**EV**	890 ± 140*	1103.4 ± 179*
**EV LSON**	155.9 ± 34* #	895.9 ± 270
**EV RSON**	184.3 ± 35* #	< 0.1 ng*#
**EV BSON**	110 ± 20*#	170 ± 70* #

*p < 0.05 vs control group

#p < 0.05 vs EV group (Student's t test)

## Discussion

Taken together, the results presented herein support the idea that PCOS induced by EV treatment results from the hyperactivity of the ovarian noradrenergic system [16,17,28], and that the SON is one of the neural pathways participating in the control of the syndrome. In addition, the effects of unilaterally sectioning the SON suggest that the lack of ovulation in PCOS animals depends on modifications in other neural mechanisms regulating ovarian functions.

The ovaries receive neural information through the SON, the ovarian plexus nerve, and the vagus nerve [29], and it has been proposed that a neural communication between the ovaries exists [30]. It is possible that the SON is one of the pathways communicating both ovaries [24]. Unilateral sectioning of the SON of adult and pre-pubertal rats results in a lack or diminution of spontaneous ovulation by the denervated ovary, and normal ovulation and compensatory ovulation by the innervated ovary [23,24]. Unilateral sectioning of the SON restored ovulation by the innervated ovary in non-ovulating rats with the lesion of the dorsal raphe nucleus (DRN), while the denervated ovary did not ovulate [31]. A similar scenario occurred in the present study, where unilaterally sectioning the SON of rats treated with Vh (ovulating) or EV (non-ovulating), resulted in spontaneous ovulation by the innervated ovary.

In rats with PCOS induced by EV-treatment, the bilateral sectioning of the SON [13,16,17] or electro-acupuncture treatment [32] restored spontaneous ovulation. According with the authors, the lower NE tone resulting from sectioning the SON or the effects of electro-acupuncture explains such results. In rats with unilateral sectioning of the SON, the lower NE concentration in the denervated ovary and high or lower levels in the innervated ovary suggest that a high NE concentration in the ovaries of rats with PCOS, induced by EV-treatment, is not the only factor explaining the PCOS.

The nerve growth factor (NGF) plays a role regulating ovarian functions [33]. The NGF contribution to the ovulatory process includes a stimulatory effect of the neurotrophin on steroidogenesis, prostaglandin E_2 _(PGE_2_) formation, and proliferative activity of thecal compartment cells [34].

In rats with PCOS induced by EV-treatment resulted in increased intraovarian synthesis of NGF and its low affinity receptor, p75 NGFR [34,35]. The ovarian denervation induced by sectioning the SON results in an increase in NFG concentration in the denervated ovary [36]. The hyperactivation of ovarian sympathetic nerves in rats with EV-induced PCOS is related to an over production of NGF suggesting that activation of this neurotrophic-neurogenic regulatory loop is a component of the pathological process by which EV induces cyst formation and anovulation in rodents [12]. According to Dissen *et al*., [34] an abnormally elevated production of NGF within the ovary suffices to initiate structural and functional alterations associated with the development of follicular cysts in the rats' ovaries. Then, it is possible that these factors contribute to the development of PCOS because, the selective blockade of NGF actions in the ovary restored ovarian function [12]. Present results show that unilaterally sectioning the SON results in ovulation by the innervated ovary and the lack of ovulation by the denervated one. We suppose that the blockade of neural communication between both ovaries could affect the NGF concentrations in the innervated-ovary, since an increase in NGF concentrations occurs in the denervated ovary [36].

In the adult rat, the acute unilateral perforation of the dorsal or ventral peritoneum results in significant and different changes for P_4_, T and E_2 _serum levels. The magnitude of the changes present depends on both the side of the perforation and the day of the estrous cycle when surgery was performed, suggesting that the neural pathways regulating hormone ovarian secretion are not the same for P4, T and E2 [37,38]. Present results suggest that changes in ovarian hormone serum levels induced by EV treatment seem to be controlled by neural signals arising from the abdominal wall and other signals.

Weiss *et al*., [39] suggested that the neural control of ovarian steroidogenesis may be excitatory, mediated by the stimulation of beta-adrenergic receptors, or inhibitory, mediated through alpha-receptors.

According to Rosa-E-Silva *et al *[17] and Lara *et al *[16], the bilateral sectioning of the SON of rats with PCOS, induced by EV-treatment to 14 or 71 day-old rats, normalized steroid ovarian hormone levels. In the present study, unilateral or bilateral sectioning of the SON of rats with PCOS induced by EV-treatment normalized vaginal estrous cycles and decreased E_2 _levels, suggesting that the neural information carried by the SON plays a stimulatory role in the regulation of estradiol secretion.

No significant changes in FSH and LH levels are observed in rats with PCOS induced by EV-treatment, suggesting that abnormalities in gonadotropins levels do not play a causal role in maintaining the polycystic condition of the ovaries [14,15]. In the present study, compared to EV treated rats, unilaterally sectioning the SON of rats with PCOS did not modify FSH or LH, supporting the idea that changes in gonadotropins levels is not fundamental for PCOS development.

## Conclusions

Taken together, our results suggest that the participation of the SON in regulating ovulation is direct in rats with PCOS. Neural signals arriving to the ovaries via the SON, and other signals originating from the peritoneum and/or the abdominal wall, play a role in the regulation of ovarian hormones secretion.

## Competing interests

The authors declare that they have no competing interests.

## Authors' contributions

LM and RD planned the experiments. LM, RL, GR, CM and RD devised the study and participated in the discussion of the results. RC and MC participated in measured hormones by RIA. All authors read and approved the final manuscript.
